# Theory, Instrumentation and Applications of Magnetoelastic Resonance Sensors: A Review

**DOI:** 10.3390/s110302809

**Published:** 2011-03-02

**Authors:** Craig A. Grimes, Somnath C. Roy, Sanju Rani, Qingyun Cai

**Affiliations:** 1 KMG2 Sensors Corporation, 430 Bailey Lane, Boalsburg, PA 16827, USA; 2 Department of Physics, Indian Institute of Technology Madras, Chennai 600036, India; E-Mail: somnath@physics.iitm.ac.in; 3 Materials Research Institute, Pennsylvania State University, University Park, PA 16802, USA; E-Mail: sanjuiitd@gmail.com; 4 State Key Laboratory of Chemo/Biosensing and Chemometrics, College of Chemistry and Chemical Engineering, Hunan University, Changsha 410082, China; E-Mail: qycai0001@hnu.cn

**Keywords:** magnetoelastic, sensor, magnetostrictive, coagulation, remote query, wireless

## Abstract

Thick-film magnetoelastic sensors vibrate mechanically in response to a time varying magnetic excitation field. The mechanical vibrations of the magnetostrictive magnetoelastic material launch, in turn, a magnetic field by which the sensor can be monitored. Magnetic field telemetry enables contact-less, remote-query operation that has enabled many practical uses of the sensor platform. This paper builds upon a review paper we published in *Sensors* in 2002 (Grimes, C.A.; *et al. Sensors* **2002**, *2*, 294–313), presenting a comprehensive review on the theory, operating principles, instrumentation and key applications of magnetoelastic sensing technology.

## 1. Introduction

Magnetoelastic sensors are, generally, ribbon-like thick-film strips made from amorphous ferromagnetic alloys, such as Fe_40_Ni_38_Mo_4_B_18_ (Metglas 2826MB). Magnetoelastic sensors with a size of approximately 4 cm × 6 mm × 25 μm are widely used as anti-theft markers. Exposure to a time-varying magnetic field produces longitudinal vibrations in these sensors that, in turn, generates elastic waves [1]. The elastic waves within the magnetostrictive magnetoelastic material generate a magnetic flux that can be detected remotely.

**Figure 1.**
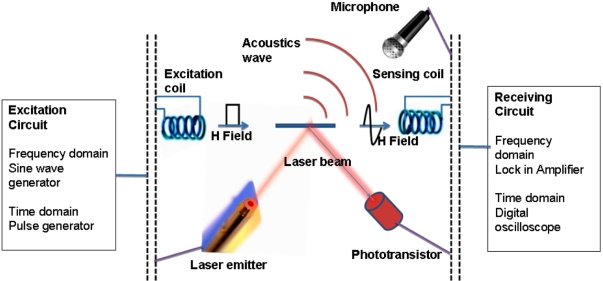
A magnetoelastic sensor strip is excited with a magnetic field. The sensor response can be detected by magnetic, acoustic or optical techniques. Reprinted with permission from [2].

As shown in Figure 1 the resonance characteristics of the sensor can be monitored by optical, acoustic or magnetic techniques. The frequency and amplitude of the longitudinal vibrations of a sensor depends on the length L, elasticity E, and density ρ of the ribbon-like thick film sensor [3]:

(1) $$f=\frac{1}{2L}\sqrt{\frac{E}{\rho }}$$

The resonance frequency changes when there is a small mass loading on the surface of the sensor. A magnetoelastic sensor of mass *m*_0_ with initial resonance frequency *f*_o_, when subjected to a mass loading (translational, uniformly applied) of Δ*m* demonstrates a decrease in resonance frequency of [3]:

(2) $$\Delta f=-f_{0}\frac{\Delta m}{2m_{0}}$$

Further, a change in the viscosity/density of the medium surrounding a sensor creates a damping effect on the sensor vibrations. The shift in resonance frequency Δ*f* is related to the viscosity *η* and density *ρ_l_* of the surrounding medium as [4]:

(3) $$\Delta f=\frac{\sqrt{\pi f_{o}}}{2\pi \rho_{s}d}\sqrt{\eta \rho_{l}}$$

where *d* is the thickness of the magnetoelastic sensor and ρ_s_ the density of the sensor.

The resonance changes in a magnetoelastic sensor due to changes in the surrounding medium forms the basis of the sensing mechanism [2,5,6]. The ability of a magnetoelastic sensor to respond to changes in the ambient has resulted in many applications involving detection and measurement of physical parameters such as pressure [7–9], temperature [10–12], liquid density and viscosity [3,13–15], fluid flow velocity [8,16], and elastic modulus of thin films [17,18]. Magnetoelastic sensors have also been used for chemical sensing through their combination with a thin, chemically sensitive over-layer, the mass of which changes upon interaction with a chemically active ambient, thereby causing a shift in sensor resonance properties. Such magnetoelastic chemical sensors have been successfully implemented, for example, in the gas-phase sensing of humidity [8,10,19], carbon dioxide [20], and ammonia [21]. In liquid, magnetoelastic sensors have been used for measurement of solution pH [11,22], and different chemical-biological agents such as glucose, avidin, ricin, endotoxin B, and E. *coli* 0157:H7 [23–27]. Recently, magnetoelastic sensors have been successfully used to analyze and quantify coagulation dynamics and platelet functions of blood and platelet-rich plasma [28–32]. The wireless, remote-query nature of the magnetoelastic sensor platform can be of tremendous advantage where a direct probe or an electrical contact with the sensing element is not feasible.

## 2. Theory of Operation

### 2.1. The Equation of Motion of a Magnetoelastic Sensor

Magnetoelastic sensors are magnetostrictive, so a strain develops in the material when subjected to a magnetic field. Magnetoelastic vibrations in a magnetoelastic sensor occur when the applied magnetic field is time varying in nature, causing the field-generated strain to vary with time thus producing a longitudinal elastic wave. The resonance frequency and amplitude of such vibrations depend not only on the sensor material but also on the surrounding medium that acts as a damping force to the sensor oscillations. Here we present the theoretical model of a magnetoelastic sensor starting from the equation of motion and deriving the resonance conditions; boundary conditions will then be applied to the basic equation to understand how a magnetoelastic sensor behaves under various conditions [33–35].

**Figure 2.**
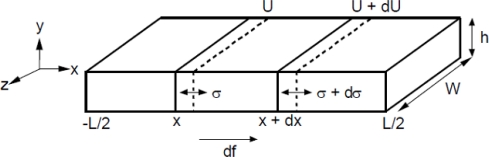
Orientation of forces acting on a magnetoelastic sensor used in deriving the equation of motion. Reprinted with permission from [33,34].

We begin by considering longitudinal elastic waves propagating along a thin metallic plate. Figure 2 shows the schematic of such a sensor. Variable *U* represents the displacement of a cross-sectional slice of the sensor from its equilibrium position *x*. *A* is the cross-sectional area and is assumed constant along the length of the sensor. Length *l* of the sensor is along the *x*-axis, the width *w* along the *z*-axis, and thickness *h* along the *y*-axis. The sensors are assumed free-floating, hence internal driving forces and displacements are symmetric about the center of mass. For this reason the origin is conveniently placed at the center of the sensor with the ends at ±*l/*2, ±*h/*2, and ±*w/*2. If we consider a small segment of the sensor, shown in Figure 2, from x to x + dx, the strain *e* in the segment may be written as:

(4) $$e=\frac{U+\mathrm{dU}-U}{x+\mathrm{dx}-x}=\frac{\mathrm{dU}}{\mathrm{dx}}$$

The internal stress (σ) at the same position is determined from the strain and the effective modulus of elasticity under applied magnetic field (*E_H_*):

(5) $$\sigma =E_{H}\frac{\mathrm{dU}}{\mathrm{dx}}$$

The net force acting on the element dx will be the change in stress over *σ* at *x* to *σ* *+* *dσ* at *x* + *dx*. This will cause an incremental change in stress *(dσ/dx)δx* and the resulting elastic force due to a change in stress will be:

(6) $$f_{\mathrm{elastic}}=A\left(\sigma +d\sigma -\sigma \right)=\mathrm{Ad}\sigma =A\frac{d\sigma }{\mathrm{dx}}\mathrm{dx}=E_{H}A\frac{d^{2}y}{{\mathrm{dx}}^{2}}\mathrm{dx}$$

Damping forces also act on the sensor. The most common is viscous damping, in which the damping force is proportional to the velocity of the object (the sensor segment) that opposes the motion. If we consider *Aρδx* to be the mass of the sensor segment that moves with a velocity *dU/dt* then the force due to viscous damping may be expressed as:

(7) $$f_{\mathrm{damping}}=D_{o}\frac{\mathrm{dU}}{\mathrm{dt}}\rho A\;\;\mathrm{dx}$$

where *D*_0_ is the damping coefficient and *ρ* is the sensor density.

Because of the metallic, conductive nature of the sensors and the presence of a time varying magnetic field, eddy current effects must also be taken into account. The damping due to eddy current effects is given by [36]:

(8) $$D_{\mathrm{eddy}}=\left[\frac{\pi^{2}h^{2}}{6\rho_{\mathrm{el}}}F\chi_{o}\chi \right]k^{2}$$

Here *F* denotes the force, χ_o_ the susceptibility of free space, χ the differential susceptibility, *ρ*_el_ the electrical resistivity, and *k^2^* the magnetoelastic coupling coefficient. The total damping is the sum of mechanical and eddy current damping:

(9) $$D=D_{o}+D_{\mathrm{eddy}}$$

The total force acting on the sensor segment *δx* is equal to the sum of the elastic and damping forces:

(10) $$E_{H}A\left(\frac{d^{2}U}{{\mathrm{dx}}^{2}}\right)\partial x-D\left(\frac{\mathrm{dU}}{\mathrm{dt}}\right)A\rho \partial x=\mathrm{Total}\;\mathrm{force}=\mathrm{ma}=A\rho \;\;\partial x\left(\frac{d^{2}U}{{\mathrm{dt}}^{2}}\right)$$

The equation of motion can therefore be written as:

(11) $$E_{H}\frac{d^{2}U}{{\mathrm{dx}}^{2}}-D_{\rho }\frac{\mathrm{dU}}{\mathrm{dt}}=\rho \frac{d^{2}U}{{\mathrm{dt}}^{2}}$$

This is similar to an equation of periodic motion, the frequency of which, in this case, is the frequency of the applied magnetic field. A general solution can therefore be written as:

(12) $$U\left(x,t\right)=\left[U_{1}\;\text{cos}\left(\beta x\right)+U_{2}\;\text{sin}\left(\beta x\right)\right]e^{j\omega t}$$

where *U*_1_ and *U*_2_ are constants and the complex wave number *β* is related to angular frequency *ω* by:

(13) $$\beta^{2}=\frac{\omega^{2}}{v^{2}}-j\omega \frac{D}{v^{2}}$$

A solution to this equation gives the value of the resonance frequency due to the applied magnetic field *H* as:

(14) $$\omega_{H}^{2}=\frac{\beta^{2}E_{H}}{\rho }$$

After applying appropriate boundary conditions (14) becomes:

(15) $$\omega_{o}=\frac{\pi }{l}\sqrt{\frac{E_{H}}{\rho }}$$

Taking into account the effect Poisson’s ratio, σ_p_, the fundamental resonant frequency becomes [37]:

(16) $$f=\frac{1}{2L}\sqrt{\frac{E_{H}}{\rho (1-\sigma )}}$$

In sensor applications the instrumentation is designed to detect this resonance frequency and corresponding resonance amplitude, and their change with ambient conditions. For Metglas^TM^ alloy 2826MB σ is approximately 0.33 [37].

### 2.2. Effect of Mass Loading on a Magnetoelastic Sensor

If a coating of mass Δ*m* is uniformly applied on the sensor surface, the density *ρ* can be replaced by (*m*_s_ + Δ*m*)/*Ad*, where *m_s_* is the mass of the sensor, *A* is the surface area, and *d* is the thickness of the sensor. Solving the equation of motion using the modified *ρ* yields a fundamental resonant frequency given by [3]:

(17) $$f=\frac{1}{2L}\sqrt{\frac{1}{1+\frac{\Delta m}{m_{s}}}\frac{{\mathrm{Ad}}_{s}}{m_{s}}\frac{E_{H}}{\rho_{s}\left(1-\sigma^{2}\right)}}=f_{o}\sqrt{\frac{1}{1+\frac{\Delta m}{m_{s}}}}$$

For small mass loadings, Equation (17) reduces to (2):

$$\Delta f=f_{\mathrm{mass}\;\;\mathrm{loaded}}-f_{o}=-f_{o}\frac{\Delta m}{2m_{s}}$$

**Figure 3.**
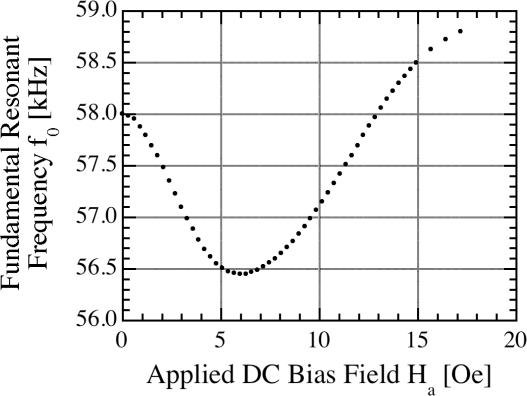
Resonant frequency of a 2826MB magnetoelastic sensor ribbon as a function of applied magnetic DC biasing field. Reprinted with permission from [18].

Figure 3 shows the fundamental resonant frequency of a magnetoelastic Metglas™ 2826MB sensor ribbon, 38.1 mm by 12.7 mm by 30.5 μm in size, as a function of the applied DC magnetic biasing field [18]. The change in resonant frequency with applied DC magnetic field seen in Figure 3 is commonly referred to as the *ΔE* effect. A change of the apparent modulus of elasticity *E*, depending on the state of magnetization, results in a change of the resonant frequency. The minimum resonant frequency occurs when the applied biasing field *H*_*DC*,min_ is close to the anisotropy field *H*_*a*_; the magnetoelastic coupling coefficient reaches a maximum at the same biasing field which is optimal for operation in that the sensor obtains the largest amplitude resonance. As the sensor is exposed to larger amplitude magnetic fields it becomes magnetically saturated with the amplitude of vibration decreasing.

In general external magnetic noise is not an issue with respect to operational accuracy, although depending upon the experimental parameters, and desired level of precision, it of course could be. As the sensors become smaller or of higher aspect ratio the optimal DC magnetic biasing field becomes larger, so variations in ambient magnetic noise have less effect. Normalized measurements, where the sensor is tracked from a given starting value, can also be performed to eliminate the effect of slowly varying changes in ambient magnetic noise.

**Figure 4.**
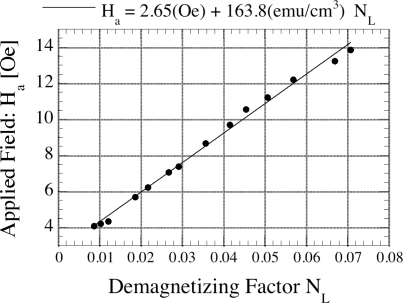
Applied magnetic DC bias field needed to reach minimal resonant frequency *vs.* demagnetizing factor for magnetoelastic sensors of varying lengths. Reprinted with permission from [18].

Figure 4 shows the influence of the shape anisotropy for sensors of different aspect ratios, and hence demagnetizing factor *N*_*L*_. The graph shows the applied DC magnetic field necessary to reach a minimal resonant frequency *vs.* the demagnetizing factor *N*_*L*_ [18] for magnetoelastic sensors of different aspect ratios. The demagnetizing factor *N*_*L*_ relates the magnetic field applied to a ferromagnetic specimen *H*_*appl*_, its magnetization *M* and internal magnetic field *H*_*in*_ by:

$$H_{\mathrm{in}}=H_{\mathrm{appl}}-N_{L}\cdot M$$

**Figure 5.**
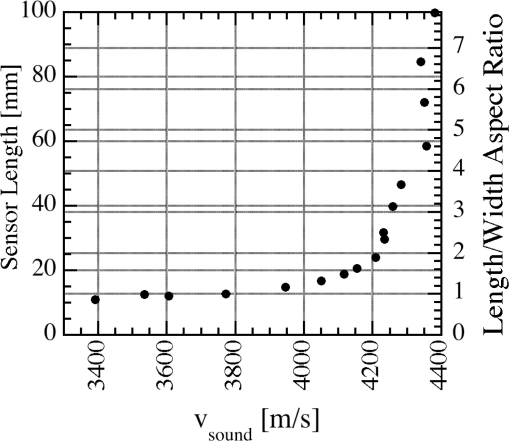
Velocity of sound in Metglas™ 2826MB sensors of 12.7 mm width at different lengths. Reprinted with permission from [18].

The linear characteristic in the data of Figure 4 indicate that, at the minimum resonant frequency, the sensors have the same state of magnetization and therefore the same sound velocity *v* independently of disturbances like interfacial stress, superimposed magnetic fields, or mechanical stresses. The descriptive operational model assumes a uniform applied field and the propagation of a uniform acoustic plane wave along the ribbon axis. Consequently, the ribbon length has to be greater than the transverse dimensions. Figure 5 shows the measured sound velocity for sensors of different lengths [18]. As can be seen from Figure 5, a length-to-width aspect ratio of 5:1 is preferred for accurate magnetoelastic resonance measurements.

## 3. Magnetoelastic Sensor Signal Detection Systems and Instrumentation

### 3.1. Time-Domain Measurement [38]

In the time-domain measurement technique the sensor is excited with a sinusoidal magnetic field impulse, commonly generated by passing current through a coil. The exponentially decaying sensor response is detected, and the resonance frequency of the sensor determined by either frequency counting or use of a Fast Fourier transform (FFT). The FFT algorithm converts the time-domain response into a frequency spectrum, and the resonance frequency is determined by identifying the peak of the spectrum. The frequency counting technique determines the resonance frequency of the sensor by counting the number of oscillations over a given period of time. Although simpler to implement, the frequency counting technique cannot quantify the quality factor of the resonance.

### 3.2. Frequency-Domain Measurement [39]

A fixed-frequency, steady-state signal is generated and the sensor response is measured at that frequency. The electronics are programmed to sweep the steady-state fixed-frequency interrogation signal over a predetermined range. The sensor resonance frequency is determined as that frequency at which the maximum response, *i.e.*, greatest sensor amplitude, is obtained.

### 3.3. Impedance De-Tuning Method [5]

A magnetoelastic sensor is inserted inside an inductive solenoid, and the impedance of the solenoid measured as a function of frequency. Since the permeability of the sensor increases significantly at resonance, a sharp peak occurs in the solenoid’s impedance spectrum at the resonance frequency of the sensor.

### 3.4. Instrumentation: Early Developments

**Figure 6.**
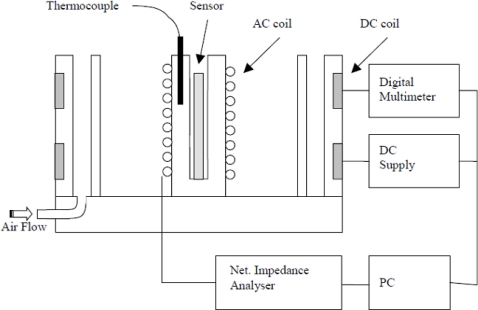
Schematic drawing of common generic components of a magnetoelastic sensing system. Reprinted with permission from [40].

Figure 6 shows a schematic drawing of components associated with a laboratory impedance-analyzer based magnetoelastic sensor instrumentation system [40]. Two sets of magnetic coils are used, one to generate the time varying interrogation signal and detect the sensor response, the other to provide a dc magnetic bias field.

**Figure 7.**
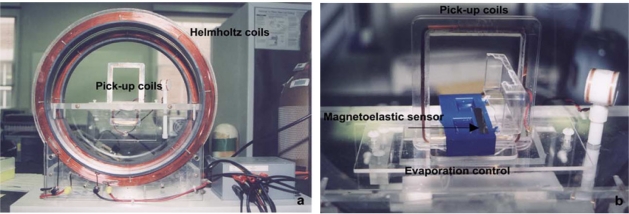
Magnetoelastic-sensing system coil design in its infancy. Shown is a side view of the Helmholtz coils used to generate the ac and dc fields, and an enlarged view of the rectangular pick-up coil with the sensor stage in the middle. Reprinted with permission from [28].

The coils generate the applied magnetic fields to excite the sensors, and the change in their impedance provides information on the sensor response. The ac and dc magnetic fields can be generated by using either two separate coils, or by using a single coil with the superimposition of both ac and dc currents. It was common for two separate coils, superimposed upon the other, to be used in the early stages of magnetoelastic sensor development; a picture of such a system used by Puckett *et al*. [30] for monitoring blood coagulation is shown in Figure 7. The ac coil is designed to surround the sensor, and the impedance of the sensor is proportional to the impedance of the empty coil. Hence it is advantageous to have a large coil inductance that would make for a large sensor response. This can be achieved by increasing number of turns in the solenoid that would enhance the inductance. However, increasing the number of turns increases parasitic capacitance between each turn and each wire layer. This parasitic capacitance leads to self-resonance of the coil. As the operating frequency of the sensor approaches the self-resonance frequency of the coil, the frequency response becomes non-linear. The magnetoelastic sensor is therefore operated at frequencies much below than the self-resonance frequency of the surrounding solenoid coil.

Since the sensor impedance is proportional to the ratio of the sensor cross sectional area to the area of the coil, the best way to improve the sensor response is to decrease the size of the solenoid coil. Decreasing the size of the coil reduces the amount of magnetic flux inside the coil, even if the applied field remains the same; if the size of the sensor is kept constant, the relative contribution of the sensor to the overall impedance increases. Reducing the coil size also reduces the length of wire necessary to make the coil, which decreases the resistive loss and parasitic capacitance, increasing the self-resonance frequency. For circular solenoids, a 20 mm long, 12.5 mm diameter solenoid has an inductance of ≈100 *μ*H at a frequency of 300 kHz.

Oftentimes in laboratory settings the impedance of the solenoid and the sensor is measured with a network impedance analyzer. An impedance analyzer, such as an HP model 8753D Network/Impedance analyzer, is connected directly to the ac-field solenoid. The network analyzer operates in a constant power mode; therefore, as the impedance of the coil changes with frequency due to the coil inductance and sensor resonance the amount of current and hence the strength of the applied magnetic field changes. In a constant power setting, since the sensor response is defined as an impedance by the ratio of induced voltage to the applied current, variation in the current and the magnitude of the ac magnetic field do not directly affect the sensor response unless the amplitude of the applied magnetic field is large enough to distort the linear behavior of sensor (*i.e.*, too large an excitation field results in multiple resonance peaks of equal magnitude). The inverse relationship between current and impedance in a constant power setting indicates that when the impedance is at a maximum the current and ac field are at a minimum. The increase in impedance caused by resonance of a magnetoelastic sensor reduces the amplitude of the applied field at peak resonance amplitude. Thus, for this measurement approach, distortion related to the amplitude of the ac magnetic field decreases during resonance and the impedance of the empty coil is used as an upper limit to the amount of applied magnetic field. Typical experiments are conducted at an analyzer drive power of 0 dBm, which gives an applied field of approximately 4 A/m for the empty solenoid at a frequency of 300 kHz.

### 3.5. Modern Microcontroller Based Instrumentation [41]

**Figure 8.**
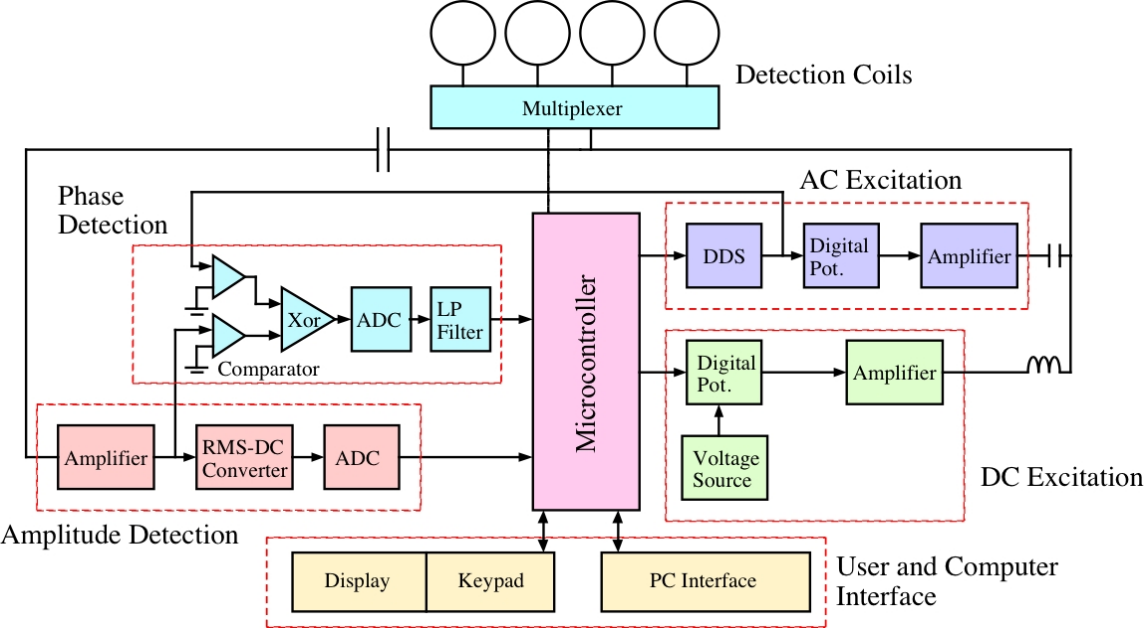
Block diagram of the microcontroller based magnetoelastic sensing system design for simultaneous monitoring of four sensors (four solenoid coils, with a sensor in a each coil). Reprinted with permission from [41].

A functional and practical magnetoelastic sensor system requires compact design, portability, ease of operation and reliability in terms of signal-to-noise ratio. To fulfill such objectives, Zeng *et al*. [41] designed and implemented a microcontroller based, single circuit board embedded system that, when interfaced with a computer, uses a single solenoid coil to characterize sensor resonance behavior in the frequency domain by obtaining the complex (magnitude, phase) impedance spectrum. Figure 8 shows the block diagram of the circuit design that electronically records and analyses the impedance spectrum of a magnetoelastic resonance sensor placed inside one of four solenoid coils. The key components of the system include:

*Microcontroller*: A DS87C520 microcontroller belonging to the 8052 microcontroller family from Dallas Semiconductor Corporation is used, chosen for its efficient bit manipulation, easy I/O interface, and large on-chip memory. It can work at frequencies up to 33 MHz; in the actual circuit a 20 MHz crystal clock is used. The microcontroller is programmed using MCS-51 assembly language.

*RS-232 Interface*: The RS-232 serial interface is used to communicate with a computer. The serial port 0 of the microcontroller is used for the interface and the Timer 2 is used to provide a baud rate of 9600.

*Direct Digital Synthesis*: A direct digital synthesis (DDS) component is used to digitally synthesize a sine wave of numerically controlled frequency from a reference clock. The DDS chip (for example, AD9832 from Analog Devices) is serially interfaced with the microcontroller sharing the 20 MHz crystal clock and provides a synthesized sine wave with a frequency resolution of 0.04 Hz.

*Multi-channel ADC*: For analog to digital conversion, a four channel 10-bit ADC chip (AD7817) is serially interfaced with the microcontroller to digitize both magnitude and phase of the sensor signal. The AD7817 has a voltage measurement resolution of about 2.5 mV when referenced to a voltage of 2.5 V.

*RMS-to-DC converter*: An RMS-DC converter (AD536A) is used for computing the true RMS value of the ac signal, and with the help of an external capacitor having a 450 kHz bandwidth converting this value into a DC signal.

*Phase detection*: The phase of the received sensor signal is measured using a phase detection circuit. Both the reference and sensor signals are first fed into comparators and the outputs are passed through a XOR gate. The output of the XOR gate is a square-wave with duty cycle proportional to the phase difference Δ*φ* between the signal and the reference signal.

**Figure 9.**
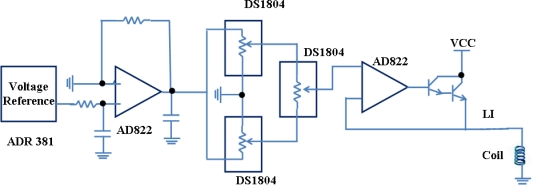
A schematic representation of the DC biasing circuit the block-diagram of which is shown in Figure 5. Reprinted with permission from [41].

*DC biasing circuit*: The dc biasing circuit supplies power to the coil to generate a dc magnetic field, using for tuning the sensor to an optimal operational point. It is designed using a combination of a voltage reference (ADR381), potentiometers (DS1804) and a large inductor (15 MH) to block the ac component. A circuit schematic is shown in Figure 9.

**Figure 10.**
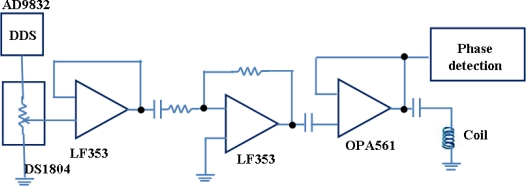
A schematic representation of the AC biasing circuit the block-diagram of which is shown in Figure 5. Reprinted with permission from [41].

*AC excitation circuit*: Figure 10 shows the ac excitation circuit used to generate the ac magnetic field that excites the sensor. To achieve this, the output of the DDS is fed into a potentiometer and amplified by an LF353 op-amp, then boosted by an OPA561 high current op-amp. A small capacitor of 1,000 pF is used to couple the ac signal to the coil.

*Receive circuit*: The receive circuit consists of an inverting amplifier with a voltage gain of 20, and a voltage follower, the output of which is sent to the RMS-DC converter for measurement of amplitude and phase.

**Figure 11.**
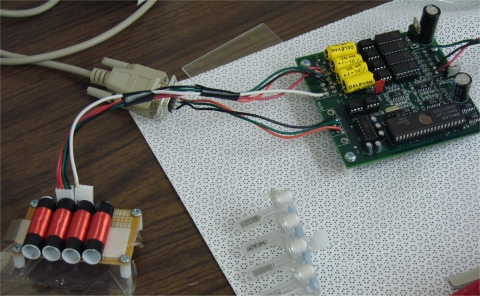
Picture of the four-channel micro-controller based magnetoelastic circuit board, with four solenoid coils shown in foreground. Reprinted with permission from [41].

**Figure 12.**
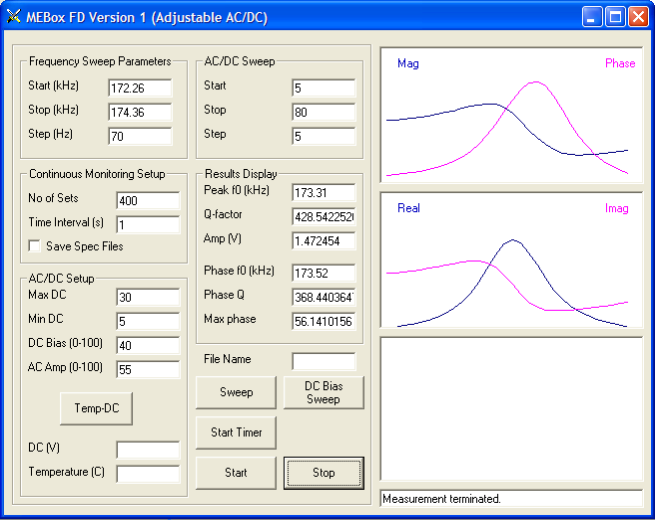
Snapshot of the graphic users’ interface (GUI) window used for single-channel magnetoelastic sensor measurements. Reprinted with permission from [41].

Figure 11 shows a four-channel magnetoelastic sensor circuit board, connected with four solenoid coils. The solenoid coils, 30 mm in length and 11 mm in diameter, are made from PTFE tubing, with 200 windings of 0.2 mm diameter insulated copper wire. In many experiments the magnetoelastic sensor strips were 12.7 mm × 6 mm × 28 *μ*m, with the sensors cut from a continuous 28 *μ*m thick Metglas 2826 ribbon of 6 mm width. The dc bias is adjusted so that such sensors have a resonance frequency of 169.7 kHz. A graphical user interface (GUI) is designed using Microsoft^®^ Visual Basic^®^ 6.0 to control experimental measurements. The GUI allows the user the digitally specify all measurement parameters, such a frequency sweep range, frequency steps, dc biasing field, and ac excitation voltage. A snapshot of the GUI window for control of a single channel measurement is shown in Figure 12. The real and imaginary parts of the sensor response, and magnitude and phase of the signal are displayed.

**Figure 13.**
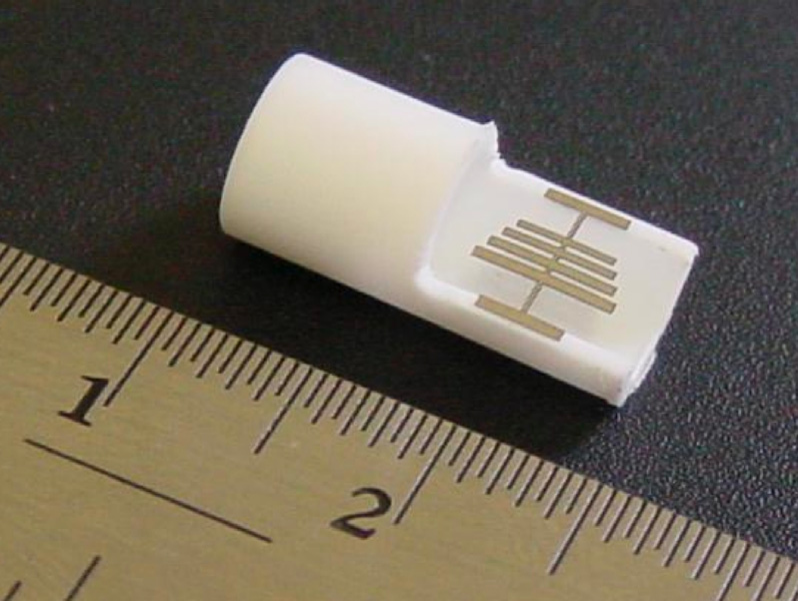
A four sensor magnetoelastic sensor array, laser-cut from a continuous ribbon. Reprinted with permission from [42].

A powerful attribute of the magnetoelastic sensor system is that the electronics can monitor multiple sensors just as easily as one. Figure 13 is a photograph of an illustrative four-element magnetoelastic sensor array, made by laser cutting of a magnetoelastic thick film.

**Figure 14.**
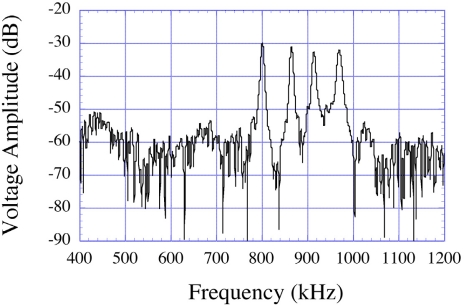
Simultaneous measurement of a four-element magnetoelastic sensor array, such as that shown in Figure 10, after a FFT conversion of captured time-domain response. Reprinted with permission from [11].

Figure 14 shows the collected data, obtained through a FFT of the time domain data, of the resonance spectrum of a similar four-element sensor array [11]. The ability to measure multiple sensors, either simultaneously or on a multiplexed basis, enables the user to cross-correlate signals and thereby remove unwanted cross-competing effects—an example of this will be shown.

## 4. Application of Magnetoelastic Sensors for the Sensing of Physical Parameters

### 4.1. Pressure Sensing

**Figure 15.**
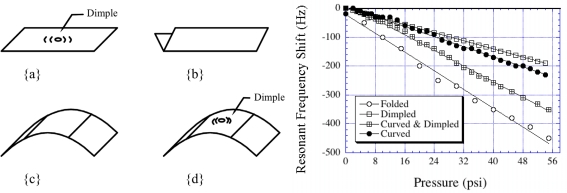
Different geometries used to stress the sensor: **(a)** a flat sensor with a dimple, **(b)** a folded sensor, **(c)** a curved sensor, and **(d)** a curved sensor with a dimple. Shift in sensor resonant frequency *vs.* pressure for the four sensor configurations. Reprinted with permission from [9].

A magnetoelastic sensor responds to changes in ambient pressure. Initial attempts in pressure measurement by Grimes *et al*. [7] involved a mounted sensor configuration, with a plexiglas support that (luckily, and inadvertently) stressed the sensor in turn generating out of plane vibrations. When the sensor was very precisely held such that no out of plane vibrations were generated, only longitudinal vibrations, the sensor demonstrated no pressure sensitivity. With a ‘slightly’ stressed sensor a linear relationship between pressure and resonance frequency was obtained due to the large surface area out-of-plane vibrations more effectively interacting with the ambient pressure. To enhance pressure sensitivity Kouzoudis and Grimes [9] suggested creating an out-of-plane vertical vibration by purposely bending the sensor, see Figure 15. The idea was to induce a stress on the magnetoelastic sensors by which the magnetoelastic energy was coupled to create a basal plane vibration; these vibrations are similar to that obtained on the surface of a vibrating drum. The resonant frequency shift of the bent sensor represented as [9]:

(18) $$\Delta f=f-f_{o}=-\frac{1}{\sqrt{3}}\frac{\sigma^{2}}{1-\sigma^{2}}\frac{m_{g}u}{k_{B}\mathrm{Td}\rho_{s}}p$$

where *p* is the pressure, σ is Poisson’s ratio which can be approximated as the ratio of the lateral to longitudinal strain, *d* is the sensor thickness, *u* is the maximum vibration amplitude, *T* is the temperature, *k*_B_ is Boltzmann’s constant, and *m*_g_ is the mass of the gas. This equation indicates that the resonant frequency decreases with increasing pressure. Theoretical estimates for a Metglas 2826MB alloy sensor predicted a frequency shift of −5.0 Hz psi^−1^ for a bent sensor, whereas the actual measurements demonstrated a frequency shift of −3.5 to −7.8 Hz psi^−1^ depending on the curvature of the sensor. Figure 15 shows the results obtained from experiments conducted with a 2826MB Metglas magnetoelastic sensor; the resonance frequency reduced linearly with pressure and the pressure dependence increased with curvature *r* of the sensor.

### 4.2. Temperature Sensing

**Figure 16.**
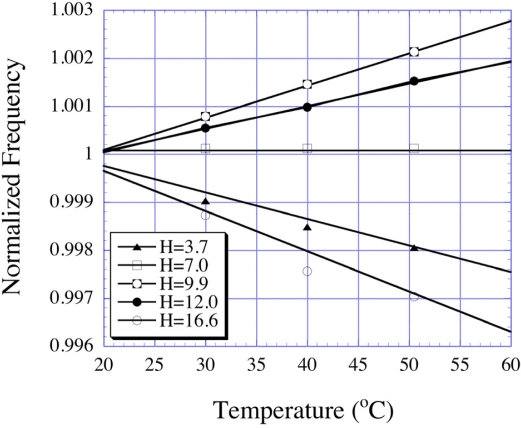
Measured resonance frequency as a function of temperature at different bias field amplitudes, 3.1 cm long sensor, normalized to the room temperature value. Reprinted with permission from [42].

The resonance frequency dependence of a magnetoelastic sensor on temperature and applied magnetic field has been express as [10]:

(19) $$f_{o}(T,H)=\frac{1}{2L(T)}\sqrt{\frac{E_{s}(T)}{\rho_{s}(T)}}{\left[1+\frac{9\lambda_{s}(T)E_{s}(T)H^{2}}{M_{s}(T)H_{k\sigma }^{3}(T)}\right]}^{-1/2}$$

where *λ*_s_ denotes the magnetostriction and M_s_ the saturation magnetization of the magnetoelastic alloy comprising the sensor. For a temperature sensor, the amplitude of the DC magnetic field H is kept constant, so that the resonance frequency shifts only with a variation in temperature. The magnetic field is optimized in a way to achieve maximum temperature sensitivity, with the temperature sensitivity, positive, negative or zero, determined by the magnitude of the applied magnetic field as shown in Figure 16 [42].

**Figure 17.**
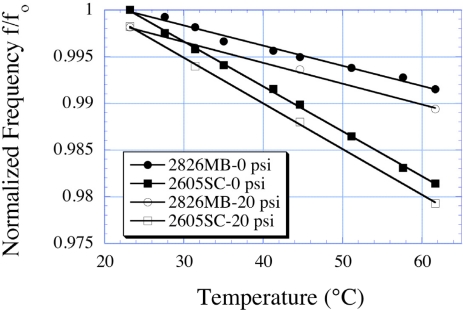
The normalized resonant frequency of a 2826MB alloy sensor, and a 2605SC alloy sensor, as a function of temperature at two different pressures. Both sensors are slightly curved, and hence demonstrate a similar, modest change in frequency with pressure. Reprinted with permission from [11].

Jain *et al*. [10] reported the temperature dependence of a 30 × 13 × 0.028 mm sensor, finding a linear frequency shift at temperatures between 20–60 °C. In subsequent work, Jain and co-workers reported the use of array type magnetoelastic sensors for the simultaneous measurements of temperature along with pressure and liquid viscosity [12,15]. Figure 17 shows, as an example, the temperature dependence of two different alloy magnetoelastic sensors at 0 psi and 20 psi. The temperature-frequency slope is unchanged with the change in pressure, allowing for temperature measurement in a changing pressure environment.

Use of magnetoelastic sensors for temperature sensing was further extended by Ong *et al*. [43], by monitoring the higher order harmonics emitted by the sensors. The technique was based upon amplitude changes in the higher order harmonics, with the ratio of different harmonics analyzed to eliminate the effect of sensor orientation relative to the sensing coils. For the tested sensors, the amplitude ratios of higher order harmonics were found to be linear between room temperature and 75 °C.

### 4.3. Measurement of Liquid Viscosity and Density

When immersed in a viscous liquid, the magnetoelastic sensor surface experiences a dissipative shear force that reduces the frequency and amplitude of vibrations. Stoyanov and Grimes [3] presented a theoretical model explaining the behavior of a magnetoelastic sensor immersed in a liquid with viscosity *η* and density *ρ_l_*. For a liquid with low viscosity, the frequency shift is given by Equation (3) repeated here for convenience:

$$\Delta f=\frac{\sqrt{\pi f_{o}}}{2\pi \rho_{s}d}\sqrt{\eta \rho_{l}}$$

**Figure 18.**
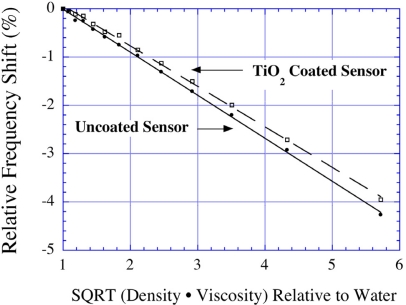
Relative frequency shifts of a bare (uncoated) and a TiO_2_ coated sensor used for the simultaneous measurement of viscosity and liquid density. Reprinted with permission from [14].

Since both viscosity and density of the liquid appear together, it was not possible to determine these variables individually with ordinary measurements. Grimes *et al*. [14] suggested the use of two magnetoelastic sensors having different degrees of surface roughness for the simultaneous determination of viscosity and density. It was assumed that, due to surface roughness, some liquid could to be trapped on the sensor surface and would act as a mass loading. In this case, the equation would be modified as:

(20) $$\Delta f=\frac{\sqrt{\pi f_{o}}}{2\pi \rho_{s}d}\sqrt{\eta \rho_{l}}-\frac{\rho_{l}\;\Delta V\;\;f_{o}}{2m_{s}}$$

where Δ*V* is the volume of the liquid trapped by the surface roughness of the sensor, and *m_s_* is the mass of the sensor. This relationship shows that the effects of liquid density and viscosity can be separated by using two sensor elements with different surface roughness that will retain different volumes of liquid Δ*V_1_* *and* Δ*V_2_*. In such a case, the difference in the change in resonance frequencies will be given as:

(21) $$\Delta f_{2}-\Delta f_{1}=\rho \frac{\Delta V_{1}-\Delta V_{2}}{2m_{s}}f_{o}$$

where *m_s_* is the mass of the sensor. Figure 18 shows the relative frequency shifts as a function of the liquid density—viscosity product for a bare (untreated) and TiO_2_ coated magnetoelastic sensor obtained by Grimes *et al*. [14]. The two sensors have different surface roughness, and hence retain different volumes of liquid on the surface. The amount of trapped liquid can be estimated with a measure of the film surface roughness that enables determination of liquid density and from there, using Equation (21), the viscosity.

### 4.4. Monitoring Fluid Flow Rate

**Figure 19.**
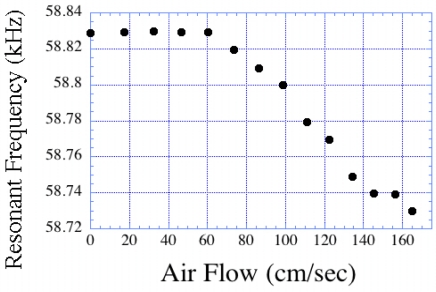
The resonance frequency shift of a magnetoelastic sensor as a function of air flow velocity. The transition to turbulent flow is not seen within this velocity regime. Reprinted with permission from [8].

When a fluid flows over the surface of a magnetoelastic sensor it creates a damping force that causes a change in the resonance frequency. Since the damping force is proportional to the flow rate, the shift in the resonance frequency also changes with a change in the flow rate. Kouzoudis and Grimes [16] reported the use of magnetoelastic sensors for measuring the flow rate of both air and water. Figure 19 shows the change in resonance frequency of a magnetoelastic sensor as a function of the air velocity it is exposed to. We note that the resonance frequency decreases in a laminar flow regime, and increases under turbulent flow.

### 4.5. Measurement of Elastic Modulus of Thin Film

When a thin film is coated onto the surface of a magnetoelastic sensor, the resonance frequency changes due to a mass loading caused by that thin film. It can be shown that the ratio of resonance frequencies are expressed as [18]:

(22) $$\frac{f_{\mathrm{coating}}}{f_{o}}=\sqrt{\left(1+\beta^{2}\right)\frac{m_{t}}{m_{c}}+\beta^{2}}=\sqrt{\left(1-\beta^{2}\right)\frac{m_{t}}{m_{c}}+\frac{m_{t}}{m_{c}}}$$

where *β* is given as:

(23) $$\beta =\sqrt{\frac{E_{c}/\rho_{c}}{E_{s}/\rho_{s}}}$$

**Figure 20.**
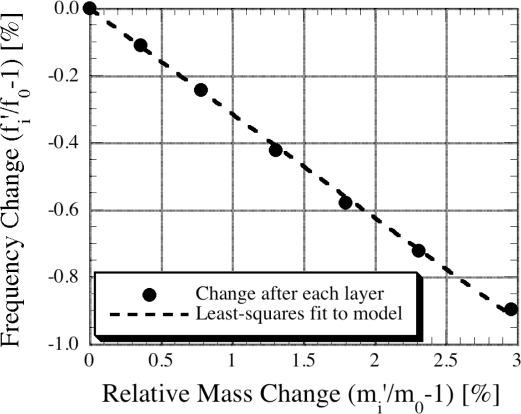
Relative frequency shift of a silver coated magnetoelastic sensor as a function of coating mass; the data was used to find the elastic constant of silver coating. Reprinted with permission from [18].

The elastic constant (Young’s modulus) of a thin film coated on the surface of a magnetoelastic sensor with a particular elastic constant can be found if the density and mass of both the coating and sensor are known. In practice, the resonance frequency and mass of a bare magnetoelastic sensor are first determined. A thin layer of coating is then applied and the mass and resonance frequency of the coated sensor is measured. To avoid errors, measurements are repeated and the value of *β* is extracted by applying a least-square fit on the collected data. Figure 20 shows the frequency shift of a silver-coated sensor plotted as a function of coating mass, as reported by Schmidt and Grimes [18]; the extracted value of Young’s modulus is 74.8 GN/m^2^, which differs from the theoretical value by only 1.6%.

## 5. Humidity and Gas Sensing

### 5.1. Humidity Measurement Using a Magnetoelastic Sensor

**Figure 21.**
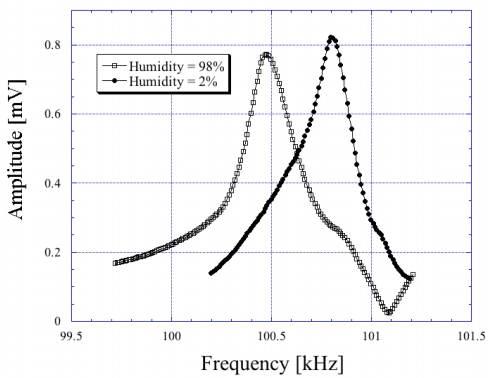
Measured frequency spectrum of magnetoacoustic humidity sensor (magnetoelastic humidity sensor excited by magnetic field and monitored acoustically) at high and low humidity levels (steady state response). Reprinted with permission from [10].

**Figure 22.**
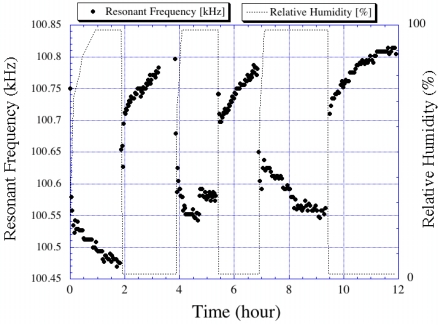
Resonance frequency of magnetoacoustic humidity sensor as a function of time as the sensor is cycled between 100% and 0% humidity ambients. Reprinted with permission from [10].

If the material coated onto a magnetoelastic sensor responds to humidity by adsorbing ambient moisture, then the resonance frequency can be expected to decrease with increasing humidity as more moisture is adsorbed leading to a greater mass loading on the sensor. In this way magnetoelastic sensors, in combination with a moisture-adsorbing layer such as TiO_2_ or Al_2_O_3_, can be used for humidity sensing. Figures 21 and 22 show the change in resonance frequency of a TiO_2_ coated sensor in response to both increasing and decreasing humidity levels, as reported by Grimes *et al*. [10,19].

The magnetoelastic sensors show excellent humidity response with a minimum hysteresis between the increasing and decreasing humidity cycles.

### 5.2. Ethylene, CO_2_ and NH_3_ Sensors

Ethylene is an organic compound that works as a plant hormone in the regulation of metabolic processes crucial for both fruit ripening and plant respiration. Traditionally gas-chromatographic systems are one of the best instruments to detect trace amounts of ethylene. However, such expensive instruments are often not practical for in-situ real-time measurements. Zhang *et al*. [44] reported the use of magnetoelastic sensors coated with Pt-TiO_2_ for the detection of ethylene. 40 mm long strips of standard 2,826 MB Metglas ribbons (6 mm × 0.028 mm) were coated with a 20 nm Pt sputtered layer followed by sol-gel deposited TiO_2_ layer. The testing was conducted in Helmholtz coil configuration with ethylene concentrations ranging from less than 1 ppm to about 60 ppm. At concentrations < 10 ppm a linear variation of the resonant frequency was observed with increasing concentration. The thickness of the TiO_2_ layer was varied from 31 nm to 155 nm, and it was found that thicker coatings resulted in a larger frequency shifts but with longer response times. A 155 nm coated sensor showed a frequency shift of 65 Hz in response to 50 ppm ethylene.

Magnetoelastic sensors can also be used for the sensing of different other gases, by a mechanism similar to that of humidity sensing, if a suitable sensing material that responds to the target gases is coated on the sensor surface. Cai *et al*. [20] reported changes in the resonance frequency of a magnetoelastic sensor coated with acrylamide and isooctylacrylate polymers of different thickness when exposed to carbon dioxide. Similarly, a sensing system for ammonia was fabricated by coating magnetoelastic sensors with a layer of poly(acrylic acid-co-isooctylacrylate) [21].

## 6. Detection of Chem-Bio Agents in Liquids

### 6.1. Magnetoelastic pH Sensor

**Figure 23.**
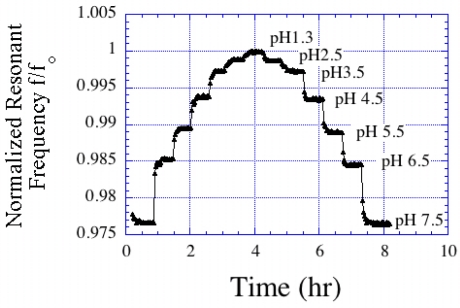
Calibration curve for a pH sensor. The frequency response is normalized to the measured value at pH = 1.3. Reprinted with permission from [11].

One of the very first applications of magnetoelastic sensors for chemical sensing was reported by Cai and Grimes [11,22], in which they coated an magnetoelastic sensor with a mass-changing pH responsive polymer that induced a change in the resonance frequency and amplitude of the sensor signal upon a change in pH. The pH responsive polymer was synthesized from 20 mol% of acrylic acid and 80 mol% of iso-octyl acrylate deposited onto the sensing strips by dip-coating. Figure 23 shows the resonance frequency shifts of a magnetoelastic sensor having different polymer layer thicknesses exposed to pH 3 and 7.5. Beyond a polymer thickness of about 4 μm, the response of the sensor was highly damped and no appreciable signal could be detected. The shift in the resonance frequency of the pH sensors decreased about 6–7% with each high/low pH cycle, most probably due to a loss of elasticity that restricted free and reversible swelling and shrinking of the polymer. The change in frequency showed almost linear behavior between pH 3 and 9 with negligible hysteresis between the cycles of increasing and decreasing pH.

Ruan *et al*. [45] further modified the pH responsive polymer coating by using spheres of poly(vinylbenzylchloride-co-2,4,5-trichlorophenyl acrylate) (VBC-TCPA), approximately 725 nm in diameter. The magnetoelastic pH sensors were fabricated by spin coating the aminated polymer spheres onto the surface of a magnetoelastic ribbon. The sensors demonstrated a linear pH response from pH 3.0 to 9.0 with a change in resonance frequency of 0.2% per pH for a 1.5 *μ*m thick coating. The measurements were found to be almost independent of background KCl concentration. Recently, Pang *et al*. [46] showed that magnetoelastic pH sensors could be used for direct measurement of body fluid acidity. A reversible and linear response was obtained with a resolution of 0.1 pH and a slope of 0.2 kHz/pH.

### 6.2. Detection of Escherichia coli O157:H7

**Figure 24.**
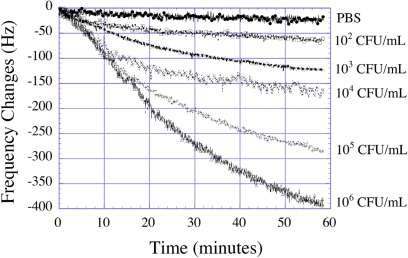
Shift in resonance frequency with time for a magnetoelastic sensor exposed to different concentrations of *E. coli O157:H7*. Reprinted with permission from [27].

*E. coli* O157:H7 is a food-borne pathogen whose detection remains one of the top priorities in the food processing industries. A common approach for *E. coli* detection is to have a binding reaction on the surface of the sensor-solution interface, in which antigen-antibody binding at the sensor surface is measured. The response of such sensors can further be enhanced by using an amplified mass immune-sorbent assay, where enzymes linked to the antibody-antigen complexes on the sensor surface catalyze to form a precipitate that results in mass loading on the sensor. Ruan *et al*. [27] applied this concept developing magnetoelastic sensor arrays for the detection of *E. coli*. A sandwich like structure consisting of *E. coli* cells and alkaline phosphatase (AP)-labeled *E. coli* antibodies formed on the surface of the sensors, and were detected by enzymatically converting 5-bromo-4-chloro-3-indolyl phosphate (BCIP) into an insoluble product that deposited on the surface of the magnetoelastic sensor thereby changing its resonance frequency. The sensing measurements were carried out with a microprocessor-based frequency counting technique that could measure the resonance frequency of different sensors with a resolution of 2 Hz over a 10 ms period. The sensor array, immobilized with antigen-antibody sandwich complexes was directly put into a vial containing 2 mL of pH 10 PBS solution with 2 mg/mL BCIP. The vial was then inserted into the testing coil for signal acquisition. Figure 24 shows the kinetic response of magnetoelastic *E. coli* sensors to the enzymatic catalytic reactions on the sensor surface with different *E. coli* concentrations. It was found that there was a steady decrease of resonance frequency with time, and the magnitude of change in frequency increased with increasing cell concentrations. This work demonstrated that bio-specific sensing interfaces can be achieved with the magnetoelastic sensor platform by immobilizing antibacterial antibodies onto a gold coated sensor element through self-assembled mono-layers resulting from cross-linking of antibody with a bi-functional binding agent.

The magnetoelastic sensor based *E. coli* detection system was later used by Huang *et al*. [47] for real-time quantification within a liquid medium. In this case, however, the magnetoelastic sensors did not need any surface functionalized with antibodies or phages. A change in solution viscosity because of the change in the nutrient concentration in the liquid medium during the growth *E. coli* caused resonance frequency shifts that was used to quantify *E. coli* concentrations on a real-time basis. It was possible to directly quantify *E. coli* concentrations of 2 × 10^2^ to 3 × 10^6^ cells/mL.

Lu *et al*. [48] proposed a modified surface functionalization technique, in which the magnetoelastic sensor was coated with a 1 *μ*m thick Bayhydrol^®^ 110 layer followed by a layer of functionalized mannose. The multivalent binding of lectin concanavalin A (Con A) to the *E. coli* surface O-antigen and mannose favored the strong adhesion of *E. coli* to the mannose modified magnetoelastic sensor. The binding was stronger because of Con A that acted as a bridge to bind *E. coli* with the surface modified sensor. In this case, the magnetoelastic sensor with *E. coli* bonded to the surface showed a resonance frequency shift that allowed a detection limit of 60 cells/mL and a linear logarithmic response range of 6 × 10^1^ to 6 × 10^9^ cells/mL.

Recently, a further modification of the surface functionalization of magnetoelastic sensors for the detection of *E. coli* has been reported by Lin *et al*. [49], in which they used chitosan-modified magnetic Fe_3_O_4_ nanoparticles (CMNPs) as the signal amplifying tags. These CMNPs were coated on a Bayhydrol^®^ layer, and at suitable pH showed strong binding to the negatively charged *E. coli* through electrostatic attraction. The *E. coli* attached to the CMNPs, and in this way were magnetically bonded to the surface of the magnetoelastic sensor resulting in a greater mass loading. This led to substantial improvement in the limit of detection with a value of 10 cells/mL. This strategy of using modified magnetic nanoparticles has also been used for the detection of polycyclic aromatic hydrocarbons (PAHs), with anthracene as the model target [50].

### 6.3. Magnetoelastic Glucose Biosensor

**Figure 25.**
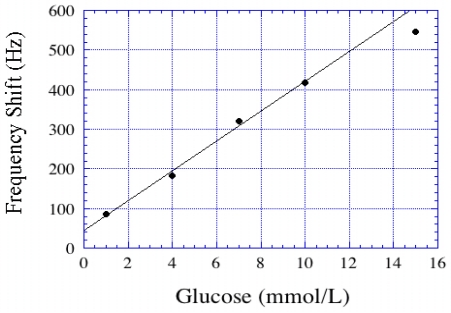
Shift in resonance frequency of a glucose oxidase (GOx) coated magnetoelastic sensor as a function of glucose concentration. Reprinted with permission from [23].

Cai *et al.* [23] reported the use of magnetoelastic sensors for glucose detection. The basic design of the glucose sensor involved deposition of a pH sensitive polymer followed by a layer of glucose oxidase (GOx). The GOx catalyzed oxidation of glucose produced gluconic acid that resulted in a reduction of pH, and subsequent shrinking of the pH responsive polymer resulting in a decrease of the polymer mass. This, in turn, caused the resonance frequency to increase. The response of the glucose biosensors was reversible and linear from 1 to10 mmol/L, see Figure 25.

The sensitivity decreased with increasing ionic strength; in 150 mmol/L of NaCl, the sensitivity decreased to 55% of the value obtained in 1 mmol/L NaCl. However, at physiological salt concentration, 0.15 mol/L, the glucose sensitivity of 0.1 mol/L was high enough for glucose detection in diabetic patients.

Pang *et al*. [51] reported magnetoelastic glucose sensors for measurement of glucose concentrations in blood plasma. At glucose concentrations of 2.5–20 mM they observed a reversible and linear response of the biosensor with a detection limit of 1.2 mM [51]. The use of magnetoelastic glucose sensors was further extended by Gao *et al*. [52] in the determination of glucose in urine samples; the shift in resonance frequency was found to be linear between 1 mM and 15 mM. The presence of acetaminophen, lactose, saccharose and galactose did not interfere with glucose detection; however, the effect of ascorbic acid had to be eliminated through cross-correlation with a pH responsive magnetoelastic sensor or by pre-adjusting the samples to pH 7.0. Initial magnetoelastic sensor measurements with 15 clinical urine samples showed glucose levels in accordance with the results obtained by a urine analyzer, which established the effectiveness of the magnetoelastic sensing technique for glucose determination.

Recently, Pang *et al*. [53] suggested the bienzyme layered assembly for glucose determination in urine samples using magnetoelastic sensors. The bienzyme layered assembly consisted of horseradish peroxidase and glucose oxidase, and has been used to detect glucose by the horseradish peroxidase-mediated oxidation of 3,3′,5,5′-tetramethylbenzidyne by H_2_O_2_ and the formation of insoluble product on the sensor surface. This insoluble product led to mass loading of the sensor and a corresponding change in the resonance frequency. This modified magnetoelastic glucose sensor showed linear response within the range of 5–50 mM, with a detection limit of 2 mM at a noise level of 10 Hz.

### 6.4. Magnetoelastic Avidin Biosensor

**Figure 26.**
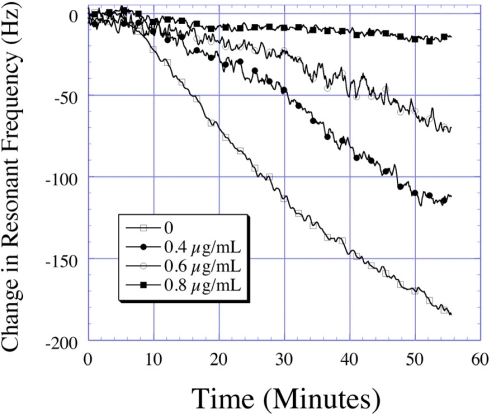
Shift in the resonance frequency with time for a PEG coated sensor as a function of different avidin concentrations. Reprinted with permission from [24].

Avidin, a glycoprotein found in white of chicken eggs, is toxic to many organisms due to its ability to deplete biotin, an essential vitamin (Vitamin H). The interaction between avidin and biotin exhibits the strongest known affinity (dissociation constant, *K*_d_ 10^15^ M^−1^) between a ligand and a protein. Following a mechanism similar to the *E. coli* detection, Ruan *et al*. [24] developed a magnetoelastic sensor for avidin detection utilizing its bio-affinity properties coupled with bio-catalytic precipitation. In this work, the surface of the magnetoelastic sensor was coated with biotinylated poly(ethylene glycol) PEG which showed specific binding characteristics to AP labeled avidin. The strong preferential binding between these two bio-chemical agents was used to quantify the avidin concentration. Bio-catalytic precipitation of BCIP through AP on the surface of the magnetoelastic sensor was used to amplify the detected mass change increasing the signal-to-noise ratio. The avidin sensing measurements were conducted with a frequency counting technique and the time dependent change in frequency monitored. Over a 60 min reaction time, the PEG functionalized magnetoelastic sensors exposed to 0.2 μg/mL avidin showed a change of 170 Hz in the resonance frequency (Figure 26). The change in frequency was found to be linear between 0.2 μg/mL and 0.75 μg/mL with a detection limit of 200 ng/mL.

### 6.5. Other Bio-Sensing Applications

Over the years, magnetoelastic sensors have been applied to the detection of many bio-chemical agents, pathogens, and bacteria. These include ricin [25], trypsin [54], *staphylococcal enterotoxin B* [26], *Staphylococcus aureus* [55], *Pseudomonas aeruginosa (P. aeru)* bacteria [56], *Mycobacterium tuberculosis* [57], *Micrococcus Luteus* [58], acid phosphatase [59], α-amylase [60], organophosphorous pesticides [61], *etc*. Simultaneous measurement of multiple bio-agents has been made possible with the development of a magnetoelastic sensor array by Ong *et al*. [62] for simultaneous detection of *E. coli O157:H7*, staphylococcal enterotoxin B, and ricin.

### 6.6. Magnetoelastic Sensor for Monitoring Milk Quality

**Figure 27.**
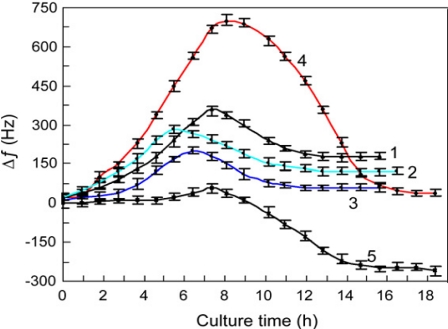
Change in resonance frequency as a function of time of a magnetoelastic sensor with the growth of different microbes in milk: (1) *E. coli*; (2) *S. epidermidis;* (3) *P. aeruginosa*; (4) *P. aureus*; (5) Blank. Reprinted with permission from [64].

Milk is one of the primary foods for people around the world, and is the source of many secondary food products such as cheese. The remote-query nature of the magnetoelastic sensors makes them an ideal platform for quality testing of milk samples. One of the first applications of magnetoelastic sensors for the milk quality determination was reported by Yang *et al*. [63], in which they analyzed the lactose component of a milk sample. The determination of lactose in a milk sample was achieved by co-immobilizing beta-galactosidase (GLA) and glucose oxidase (GOx) and catalase onto a magnetoelastic sensor that was pre-coated with a layer of pH sensitive polymer. The GLA-catalyzed hydrozylation of lactose produced beta-d-glucose that was oxidized to gluconic acid with GOx, resulting in a change of pH of the system. The corresponding change in the resonance frequency was found proportional to the lactose concentration range of 2 mM to 16 mM with a detection limit of 0.72 mM. Huang *et al*. [64] further extended the use of magnetoelastic sensors for milk testing with the monitoring of *S.aureus* bacterium-considered one of the primary agents causing milk spoilage. Figure 27 shows the growth curves of *S. aureus* and other bacteria in milk. It was shown that the magnetoelastic sensors kept inside a hermetically sealed container could detect the change in milk viscosity caused by growth of the bacterium allowing remote-query monitoring of the milk quality. By tracking the change in resonance frequency with changing viscosity, it was possible to directly quantify *S. aureus* concentrations of 10^3^ to 10^7^ cells/mL. Recently, Gao *et al*. [65] reported the use of magnetoelastic sensors to detect penicillin G in milk. A magnetoelastic sensor pre-coated with a pH sensitive polymer was further coated with a film containing bovine serum albumin (BSA) and penicillinase. The penicillinase catalyzed hydrolysis of penicillin G changed the pH of the medium that in turn changed the resonance frequency of the magnetoelastic sample. Under optimum operating conditions, the system showed a linear response between 1.9 mM and 5.0 mM with the limit of detection of 1.3 mM. The immobilized penicillinase on magnetoelastic sensors was found to be very stable and provided good reproducible signal after regeneration up to 30 times with a relative standard deviation lower than 9%. Good recoveries and precision were obtained when spiked raw milk samples were analyzed.

### 6.7. Magnetoelastic Sensor for Lipoprotein Detection

**Figure 28.**
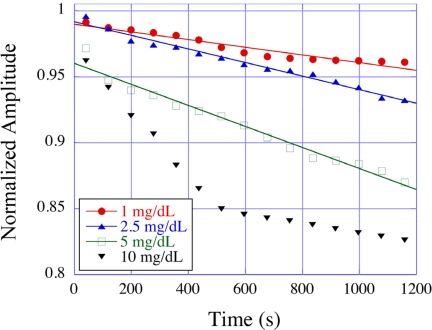
Effect of low-density lipoprotein (LDL) concentrations on the time dependent normalized amplitude of an magnetoelastic sensor immersed in LDL solution. Reprinted with permission from [66].

Lipoprotein particles are the main carriers of cholesterol in the human blood and play a key role in cholesterol transfer and metabolism. Recognition of the importance of cholesterol as a strong risk factor for coronary artery disease has resulted in great interest in techniques to rapidly and accurately quantify lipoprotein fractions in the blood stream. Feng *et al.* [66] reported the use of magnetoelastic sensors for the detection of lipoprotein particles. The measurement was based on the selective bio-chemical reaction between a precipitator (dextran sulfate, sodium salt and divalent metal Mg^2+^) and lipoproteins, producing insoluble precipitates that deposited onto the surface of a magnetoelastic sensor to change its resonance frequency and amplitude. Figure 28 shows the effect of LDL concentrations on the time dependent normalized amplitude of magnetoelastic sensors. In response to a 10 mg/dL LDL solution the sensors showed a change of approximately 20% in resonance amplitude, while experiments with HDL solutions under similar conditions revealed only a 4% change in the resonance amplitude. Magnetoelastic sensing measurements involving liquid/solid interfaces often show low signal-noise ratios and lack of repeatability due to the formation of unwanted and random formation of microscopic air bubbles on the hydrophobic metallic surface. To avoid unwanted bubble formation and increase the signal-noise ratio and measurement reliability Feng *et al*. [67] suggested using a thin layer of ZnO chemically deposited onto the surface of magnetoelastic sensors, turning the surface of the magnetoelastic sensor from hydrophobic to hydrophilic thereby increasing the wetting of the surface. In comparison to the bare, hydrophobic sensors, the standard deviation of the resonance amplitude in ZnO coated sensors decreased substantially, ranging from a 27% decrease for bovine blood to a 67% decrease for saline. These results showed that a hydrophilic coating on magnetoelastic sensor surfaces greatly enhances the repeatability and reliability of liquid measurements.

## 7. Magnetoelastic Sensors for Monitoring Blood Coagulation

**Figure 29.**
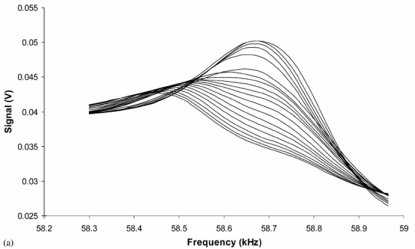
Frequency scan over time for a magnetoelastic sensor with a drop of blood on its surface; as the blood droplet clots both the resonance frequency and amplitude are reduced. Reprinted with permission from [28].

By monitoring the frequency and amplitude changes of a magnetoelastic sensor immersed in a blood sample the clotting time and strength can be determined on a remote query basis. Application of magnetoelastic sensor technology for the determination of blood clotting kinetics was first reported by Puckett and co-workers [28]. The relatively low-cost of magnetoelastic sensor ribbons enables their use as disposable sensors. The clot sensing experiments were performed in a Helmholtz coil configuration using 6.35 mm × 38.1 mm × 0.03 mm strips of 2,826 MB Metglas ribbons. Horse blood samples were collected in blood collection tubes at a University of Kentucky research farm and were tested within 4 hours of sampling. A frequency sweep on a bare sensor was first performed to indentify the resonance frequency; the sensor was then immersed in liquid having viscosity and density identical to that of blood to determine the sensor operating frequency under clotting conditions. A 10 *μ*L drop of blood was placed onto a sensor and a frequency scan was performed over a period of time. Both the frequency and amplitude were found to decrease with time; however, for obtaining a clotting profile, the time dependent signal amplitude was plotted because of a greater signal-to-noise ratio. Figure 29 shows the frequency-amplitude scans over time of a magnetoelastic sensor with a drop of blood on its surface. The time dependent change in the frequency spectra provides considerable information on the blood clotting characteristics.

**Figure 30.**
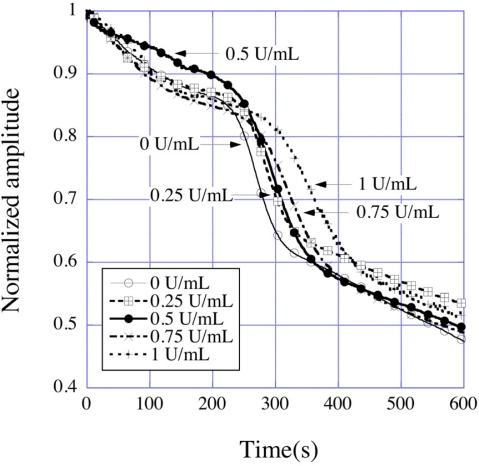
Time dependent normalized amplitude of the resonance peaks of magnetoelastic sensors immersed in blood containing different heparin concentrations. Reprinted with permission from [29].

Zeng *et al*. [29] reported the determination of activated clotting times of bovine blood at different heparin concentrations. Heparin works as an anticoagulant and increases the clotting time of blood, widely used in patients undergoing cardiac surgery. Bovine blood samples were obtained from Lampire Biological Laboratories (Ottsville, PA, USA). The obtained blood was citrated to avoid coagulation; a citrate reversal technique was therefore applied to regain normal clotting behavior of the blood. A bare sensor was first put into a small glass vial, into which a small volume of blood (1 mL) was then added; the time dependent change in resonance amplitude was measured by placing the vial in a solenoid coil such as those seen in Figure 11. The time dependent normalized amplitude of resonance peaks for the magnetoelastic sensors immersed in blood with different heparin concentrations is shown in Figure 30.

**Figure 31.**
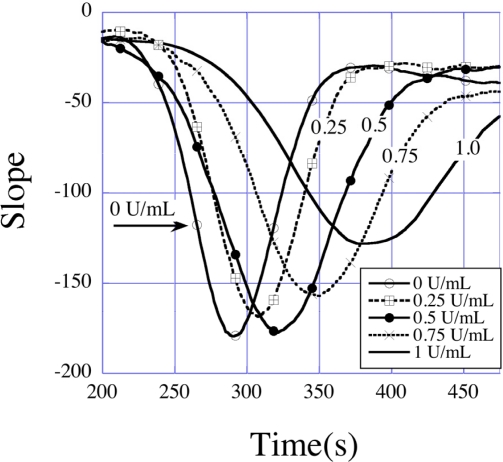
Rate of change of normalized amplitudes (curve slopes in Figure 27) showing the maximum clot rate (minima points) and corresponding clotting times (time of minima). Reprinted with permission from [29].

Three distinct regions seen in the amplitude curve represent three different processes of the coagulation dynamics of blood. The first region, where the amplitude decreases slowly with time, is the beginning of the clotting process, where the coagulation cascade is first activated. The second region is characterized by the onset of the coagulation process that results in a sharp decrease in the sensor amplitude; the actual clot formation takes place during this time, which leads to solidification of blood resulting in a severe damping of the sensor vibrations. This is followed by a third and final region, in which the coagulation process comes to an end and the decrease in amplitude slows. The second region, where the amplitude decreases at the fastest rate contains dynamic information on the actual clotting time, which can be found by plotting the slopes of the amplitude-time curves as shown in Figure 31, where the effect of heparin in delaying clot formation is clearly seen.

**Figure 32.**
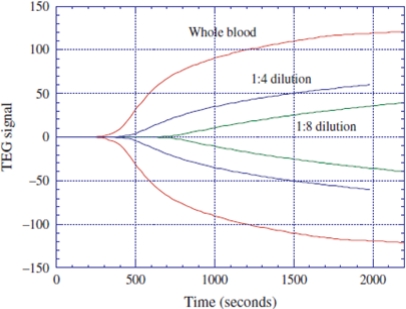
TEG-like profiles of blood samples obtained with an magnetoelastic sensing system. Reprinted with permission from [30].

Roy *et al*. [30] demonstrated that it is possible to simultaneously perform thromboelastograph (TEG) analysis and measure the erythrocyte sedimentation rate (ESR). TEG analysis and the determination of ESR are important for the detection of hemophilia, von Willebrand disease, polymyalgia rheumatica, temporal arteritis, anemia, *etc*. The magnetoelastic sensor based TEG and ESR profiler involved four sensors elements, two each for TEG and ESR, all housed in a cartridge, to simultaneously measure both clotting characteristics and settling rates. The ESR sensors are placed horizontally to maximize blood-settling effects, whereas the TEG sensors are placed vertically (on their sides) to eliminate any settling effect. To obtain a TEG profile from a blood sample, a time dependent resonance amplitude data is first taken. A TEG profile is derived from a clotting curve obtained with a magnetoelastic sensor by simply having a mirror image of the clotting curve. Figure 32 shows the TEG profiles obtained with a magnetoelastic sensing setup for blood samples with different dilutions; TEG profiles for blood samples with similar dilutions obtained from a standard TEG system (Haemoscope TEG) are shown for comparison. Similarly, an ESR profile can be derived from the settling pattern obtained with a magnetoelastic sensor by obtaining the rate of change in amplitude with time.

**Figure 33.**
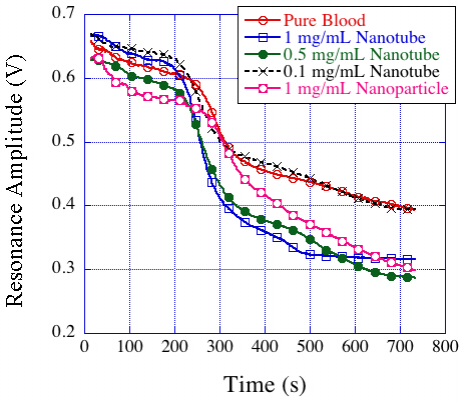
Resonance amplitude *vs.* time profiles of magnetoelastic sensors immersed in TiO_2_ nanotube-mixed blood samples. The addition of TiO_2_ nanotubes to the blood samples results in a greater clot strength and faster rate of clot formation. Reprinted with permission from [68].

Mixing bio-compatible TiO_2_ nanotubes with blood was shown by Roy *et al*. [68] to increase clot strength and the rate of blood clot formation. These experiments were conducted with bovine blood mixed with TiO_2_ nanotube powder having concentrations ranging from 0.1 mg/mL to 1 mg/mL. The resonance amplitude-time plots (Figure 33) of TiO_2_-blood samples subjected to activated clotting protocol showed a larger change in slope with higher nanotube concentrations, thereby indicating stronger clot formation.

**Figure 34.**
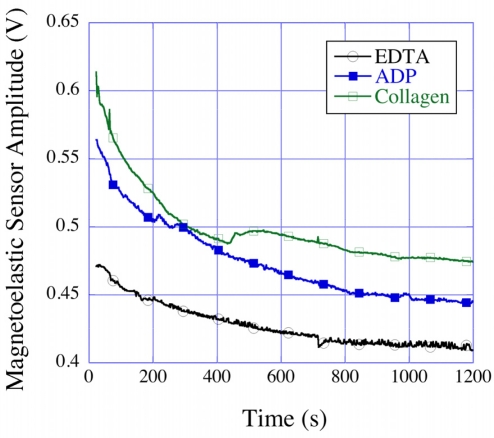
Resonance amplitude *vs.* time plots of EDTA treated whole blood samples mixed with ADP and collagen platelet activators. Reprinted with permission from [31].

Platelets play an important role in clot kinetics, in which the activation and aggregation of functional platelets are responsible for initiating the clotting cascade. The estimation of functional platelets and their ability to form aggregates is therefore crucial for diagnosis of many blood related diseases and hemostatic disorders. The standard methods for measuring the platelet aggregation and for the estimation of platelets counts require isolation of the platelets from whole blood that leads to deterioration of platelet function over time. In addition, the methods such as optical platelet aggregometry require a relatively large volume of blood/PRP. Roy *et al*. [31] used the magnetoelastic sensing platform for studying platelet aggregation by activating the whole blood/PRP samples with ADP and collagen activators, which are known to activate functional platelets and induce aggregation. Figure 34 shows the time dependent resonance amplitude data from whole blood samples mixed with different platelet activators. A greater decrease in amplitude in both ADP and collagen activated samples compared to normal (EDTA) samples was observed. This was caused by the activated platelets forming aggregates and settling onto the magnetoelastic sensor strips resulting in a mass loading and reduction in the vibration amplitudes. To confirm the measurements, platelet count tests were performed with a HemaVet^®^ 950 system on a parallel set of EDTA and ADP mixed samples prepared from the same batch of blood, which showed platelet activation and aggregation in ADP activated samples. These initial studies showed that the magnetoelastic sensing platform can be successfully used as an initial diagnostic tool for platelet aggregation tests [32].

## 8. Conclusions

Magnetoelastic sensing technology offers a versatile platform for many physical, chemical and biological sensing systems. The use of a magnetic field enables remote-query operation and therefore the technique can easily be used in wide ranging applications such as pressure and temperature monitoring, pH measurement of a solution, sensing of different gases, monitoring blood coagulation kinetics, *etc*. The dependence of the resonance frequency and amplitude of the elastic vibrations on ambient conditions ensures high sensitivity of the magnetoelastic sensing systems. Thin layers of selected polymers or oxide materials on the surface of the magnetoelastic sensors enable selective adsorption of the target component from the ambient, a property that enables use of the sensor platform for chem/bio detection. Over the last ten years significant progress has taken place in the development of both theoretical modeling and application of the magnetoelastic sensing systems. The bulky instrumentation used in the early stages of the technology development has been transformed to a microcontroller based circuit-board design that has resulted in a highly portable, efficient, and easy-to-use system. This, along with the core advantages of contact-less operation and inexpensive sensor material have made the magnetoelastic sensing platform a potential candidate for many future sensing applications.
